# Real-world effectiveness of Anti-CGRP monoclonal antibodies compared to OnabotulinumtoxinA (RAMO) in chronic migraine: a retrospective, observational, multicenter, cohort study

**DOI:** 10.1186/s10194-024-01721-6

**Published:** 2024-02-02

**Authors:** Licia Grazzi, Riccardo Giossi, Danilo Antonio Montisano, Mattia Canella, Marilena Marcosano, Claudia Altamura, Fabrizio Vernieri

**Affiliations:** 1 Headache Center, Neuroalgology Department, Fondazione IRCCS Istituto Neurologico Carlo Besta, Via Celoria, 11, Milan, 20133 Italy; 2 Poison Control Center and Clinical Pharmacology Unit, Grande Ospedale Metropolitano Niguarda, Piazza Ospedale Maggiore 3, Milan, 20162 Italy; 3 Department of Research and Clinical Development, Fondazione IRCCS Istituto Neurologico Carlo Besta, Via Celoria, 11, Milan, 20133 Italy; 4 Department of Medical Biotechnology and Translational Medicine, Postgraduate School of Clinical Pharmacology and Toxicology, Università degli Studi di Milano, Via Vanvitelli, 32, Milan, 20129 Italy; 5 Neuroimmunology and Neuromuscular Diseases Unit, Fondazione IRCCS Istituto Neurologico Carlo Besta, Via Celoria 11, Milan, 20133 Italy; 6 Fondazione Policlinico Universitario Campus Bio-Medico, Via Alvaro del Portillo, 200, Roma, 00128 Italy; 7 Department of Medicine and Surgery, Università Campus Bio-Medico di Roma, Via Alvaro del Portillo, 21, Roma, 00128 Italy

**Keywords:** Migraine, Erenumab, Galcanezumab, Fremanezumab, Onabotulinumtoxin

## Abstract

**Background:**

Chronic migraine (CM) is a disabling condition with high prevalence in the general population. Until the recent approval of monoclonal antibodies targeting the calcitonin gene-related peptide (Anti-CGRP mAbs), OnabotulinumtoxinA (BoNT-A) was the only treatment specifically approved for CM prophylaxis. Direct comparisons between the two treatments are not available so far.

**Methods:**

We performed an observational, retrospective, multicenter study in Italy to compare the real-world effectiveness of Anti-CGRP mAbs and BoNT-A. Patients with CM who had received either treatment according to Italian prescribing regulations were extracted from available clinical databases. Efficacy outcomes included the change from baseline in monthly headache days (MHD), MIgraine Disability ASsessment test (MIDAS), and monthly acute medications (MAM) evaluated at 6 and 12 months of follow-up. The primary outcome was MHD change from baseline at 12 months. Safety outcomes included serious adverse events (SAE) and treatment discontinuation. Unadjusted and adjusted models were used for the analyses.

**Results:**

Two hundred sixteen potentially eligible patients were screened; 183 (86 Anti-CGRP mAbs; 97 BoNT-A) were included. One hundred seventy-one (80 Anti-CGRP mAbs; 91 BoNT-A) and 154 (69 Anti-CGRP mAbs; 85 BoNT-A) patients were included in the efficacy analysis at 6 and 12 months of follow-up, respectively. Anti-CGRP mAbs and BoNT-A both resulted in a mean MHD reduction at 6 (-11.5 and -7.2 days, respectively; unadjusted mean difference -4.3; 95%CI -6.6 to -2.0; *p* = 0.0003) and 12 months (-11.9 and -7.6, respectively; unadjusted mean difference -4.4; 95%CI -6.8 to -2.0; *p* = 0.0002) of follow-up. Similar results were observed after adjusting for baseline confounders. Anti-CGRP mAbs showed a significant MIDAS (-31.7 and -19.2 points, *p* = 0.0001 and *p* = 0.0296, respectively) and MAM reduction (-5.1 and -3.1 administrations, *p* = 0.0023 and *p* = 0.0574, respectively) compared to BoNT-A at 6 and 12 months. No SAEs were reported. One patient receiving fremanezumab discontinued treatment due to arthralgia. Treatment discontinuations, mainly for inefficacy, were comparable.

**Conclusion:**

Both Anti-CGRP mAbs and BoNT-A were effective in CM patients with Anti-CGRP mAbs presenting higher effect magnitude, with comparable safety. Still, BoNT-A remains a valuable option for CM patients with contraindications to Anti-CGRP mAbs or for frail categories who are candidates to local therapy with limited risk of systemic administration.

**Graphical Abstract:**

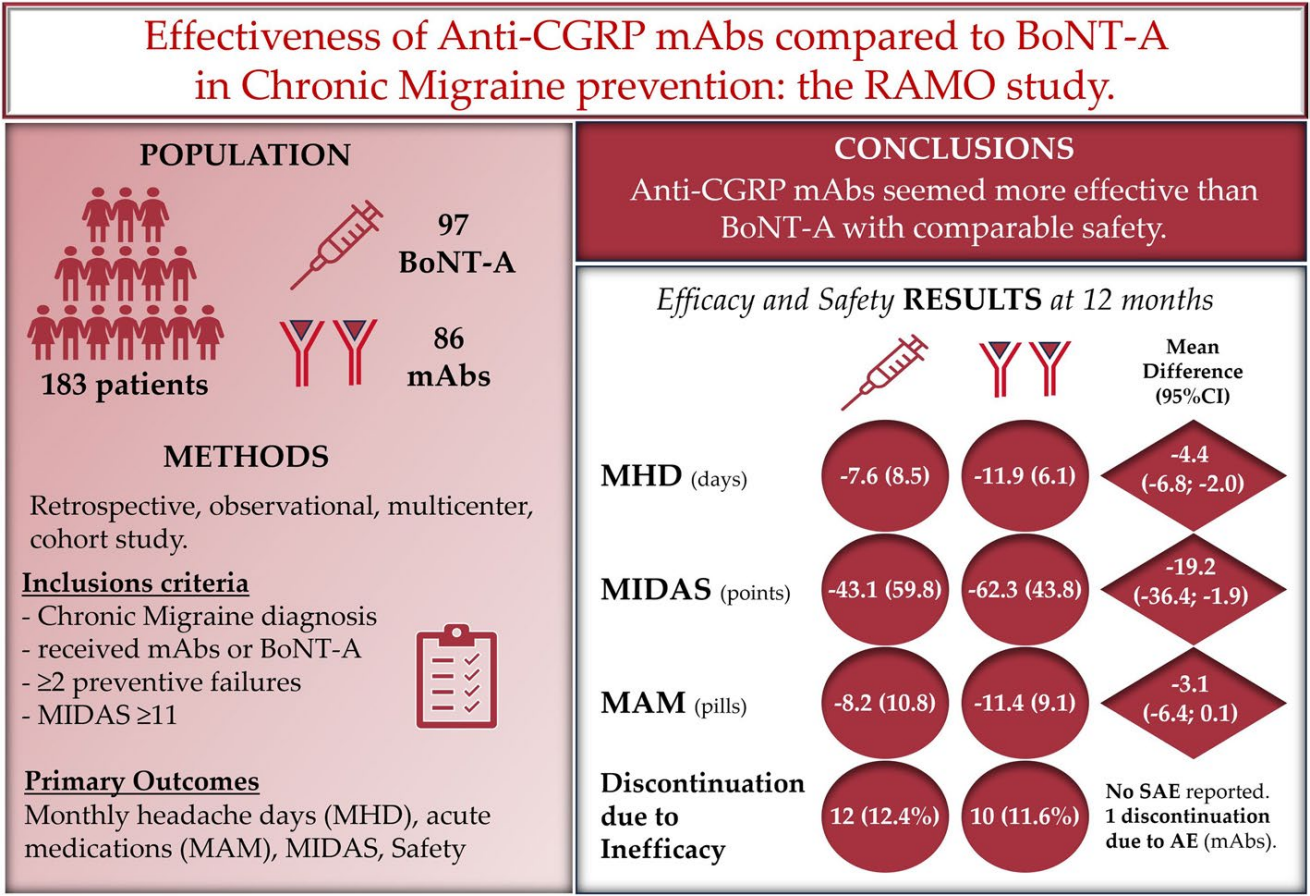

**Supplementary Information:**

The online version contains supplementary material available at 10.1186/s10194-024-01721-6.

## Introduction

Chronic migraine (CM) is a disabling condition that affects up to about 5% of the general population [1, 2]. Less common than episodic migraine, CM is associated with greater headache-related disability, higher impact on physical, social, and occupational functioning, and worse health-related quality of life [3]. CM can be associated with medication overuse headache (MOH), which makes its management more complicated [4].

The goal of CM treatment is the prevention of migraine attacks with prophylactic treatment, thereby reducing headache frequency, severity, and associated disability and decreasing reliance on acute treatment, which may be contributing to concurrent MOH [3]. Several pharmacological and non-pharmacological preventive treatments are available, but few are based on good-quality evidence of clinical efficacy [4]. One of these is intramuscular onabotulinumtoxinA (BoNT-A). However, the need for special training to administer it and treatment administration costs, which are higher than standard doses of the two first-line prophylactic treatments, propranolol and topiramate, have limited the wide use of BoNT-A in the clinical practice [5, 6]. BoNT-A effect is thought to be achieved through the blockage of sensory pain signals to the central nervous system, thereby promoting a reduction in peripheral and central sensitization. In 2010, the US Food and Drug Administration approved BoNT-A for the treatment of CM [7], based on the findings of the Phase III Research Evaluating Migraine Prophylaxis Therapy (PREEMPT) trials [8]. Subsequent studies and analyses confirmed BoNT-A safety and efficacy [9–14]. Thus, guidelines recognized its role in CM therapy [5]. BoNT-A was the only treatment approved for CM prevention in the European Union until the recent approval of anti-calcitonin gene-related peptide (CGRP) monoclonal antibodies (mAbs) [3, 7, 15]. Anti-CGRP mAbs, namely erenumab, galcanezumab, fremanezumab, and eptinezumab, block the CGRP pathway, being the first preventive treatments specifically designed for migraine and are generally characterized by a good safety profile [16–30]. Subsequent studies confirmed these results, and they resulted effective in treatment-resistant patients [31–33].

Major drawbacks of Anti-CGRP mAbs treatment are the lack of long-term data from randomized trials and elevated costs. For this reason, regulatory agencies and guidelines made them a second or third-line option for CM for some years, while recent guidelines endorsed Anti-CGRP mAbs also as first-line therapies [34, 35]. While both BoNT-A and Anti-CGRP mAbs share available evidence supporting their use for CM prophylaxis, direct comparisons of the two treatments are still limited [36]. Head-to-head trials are lacking, and placebo-controlled trials are not directly comparable. A systematic review showed that Anti-CGRP mAbs were slightly better than BoNT-A in efficacy and safety [37]. A meta-analysis argued that although Anti-CGRP mAbs showed some advantages in reducing migraine days and a possibly small advantage in causing fewer adverse events (AE), BoNT-A might be preferentially selected owing to its cost-effectiveness profile [4]. That is why we aimed to perform a real-world comparison of Anti-CGRP mAbs and BoNT-A to evaluate their effectiveness and safety, especially in the long term.

## Methods

### Study design and population

The Real-world effectiveness of Anti-CGRP Monoclonal antibodies compared to OnabotulinumtoxinA (RAMO) study is an observational, retrospective, multicenter, cohort study conducted in two hospital centers in Italy: Fondazione IRCCS Istituto Neurologico Carlo Besta (Milan) and Fondazione Policlinico Campus Bio-Medico (Rome).

We included male or female patients followed at participating centers with a diagnosis of CM according to the ICHD-3 definition, 2 or more oral preventive treatment failures, who received either Anti-CGRP mAbs or BoNT-A per Italian prescribing criteria, having 18 to 65 years at the time of treatment initiation, having a MIgraine Disability ASsessment (MIDAS) questionnaire of 11 or more before treatment initiation, and with at least 6 months of follow-up while on therapy. Patients who received BoNT-A for at least 6 months and received no Anti-CGRP mAbs for at least 12 months after BoNT-A could be included only in the BoNT-A arm. Patients who received Anti-CGRP mAbs and BoNT-A sequentially or contemporarily in their clinical history and those with concomitant severe psychiatric conditions that could interfere with study assessments upon clinician judgment were excluded.

Patients were selected from already existing anonymous records available at participating centers. Investigators checked individual patients for inclusion and exclusion criteria. For patients included in the study, available data were entered in a dedicated database realized with REDCap (Research Electronic Data Capture), hosted by Fondazione IRCCS Istituto Neurologico Carlo Besta. Collected variables included age, sex, medical history and comorbidities, concomitant medications, migraine duration, presence of MOH, previous migraine medications, and study outcome data. Since CM patients could present headache days with characteristics similar to those of tension type headache (i.e., mild continuous pain, without any accompanying symptoms typical of migraine episodes), which could be of clinical relevance and could be often associated in CM patients with MOH, the presence of tension-like symptoms (TLS) was also collected. We followed the STrengthening the Reporting of OBservational studies in Epidemiology (STROBE) statement to realize this study (Supplementary material S1).

### Study outcomes

The primary efficacy outcome of the study was to compare the monthly headache days (MHD) difference from baseline to 12 months between Anti-CGRP mAbs and BoNT-A groups. Secondary efficacy outcomes included the MHD difference from baseline to 6 months, the difference in responders at 50%, 75%, and 100% at 6 and 12 months (i.e., patients achieving a reduction of their MHD of 50%, 75%, and 100% from baseline, respectively), the difference in monthly acute medications (MAM) counted as single drug administrations (as the number of tablets or capsules), and the MIDAS difference from baseline at 6 and 12 months. MHD, MAM, and MIDAS scores were originally collected from migraine diaries in use as clinical practice at the two centers. The MIDAS is a self-reported questionnaire evaluating migraine-related disability and its impact on working, school, household, social, and leisure activities in the last 3 months. It is graded in 4 steps: little or no disability (score 0–5), mild disability (score 6–10), moderate disability (score 11–20), and severe disability (score ≥ 21) [38]. Per Italian prescribing and reimbursement criteria, a patient must have a MIDAS score of 11 or more to receive Anti-CGRP mAbs. Safety outcomes included all serious adverse events (SAE) and treatment discontinuation due to adverse events (AE) or other reasons at any time point from baseline to the end of the study. AEs were coded using the Medical Dictionary for Regulatory Activities Terminology (MedDRA) and graded according to the Common Terminology Criteria for Adverse Events (CTCAE) v5.0.

### Statistical analysis

To address the primary aim of the study (i.e., compare the 12-month change from baseline in MHD), based on the hypothesis of a mean difference between the two treatment arms of 3.2 days with a standard deviation of 7.9 days and a 10% of patients with missing outcomes or drop out, a sample of 170 (85 vs. 85) patients would have guaranteed a power of 0.8 with a 0.05 level of significance with a one-sided test. Continuous variables were described as mean and standard deviations (SD) or mean differences with 95% confidence intervals (95%CI). Categorical variables were described as counts and percentages. A comparison of baseline variables was performed with t-test, Wilcoxon rank sum test, chi-squared test, or Fisher’s exact test as applicable. The primary analysis was based on the one-sided t-test. For secondary outcomes, t-test or Mann-Whitney test, Chi-squared or Fisher exact test, and analysis of variance (ANOVA) or a corresponding non parametric analysis were used, as appropriate. We implemented an ANOVA model to evaluate the effects of significant baseline confounding variables. Marginal mean difference for the change from baseline in MHD, MIDAS, and MAM with 95%CI was estimated from the model. We also performed a sensitivity analysis using propensity score matching on significantly different baseline variables. A logistic regression model adjusted for the same confounding baseline variables was used to estimate odds ratios (OR) with 95%CI for response. Efficacy analyses were performed in a per-protocol fashion on patients who actually received the treatment and with available follow-up data. No imputation for missing data was performed. The safety analysis was descriptive with events reported as counts and percentages. An exploratory analysis comparing the different Anti-CGRP mAbs was performed. STATA 15 (StataCorp LLC) software was used for all analyses.

## Results

### Study population characteristics

**Fig. 1:**
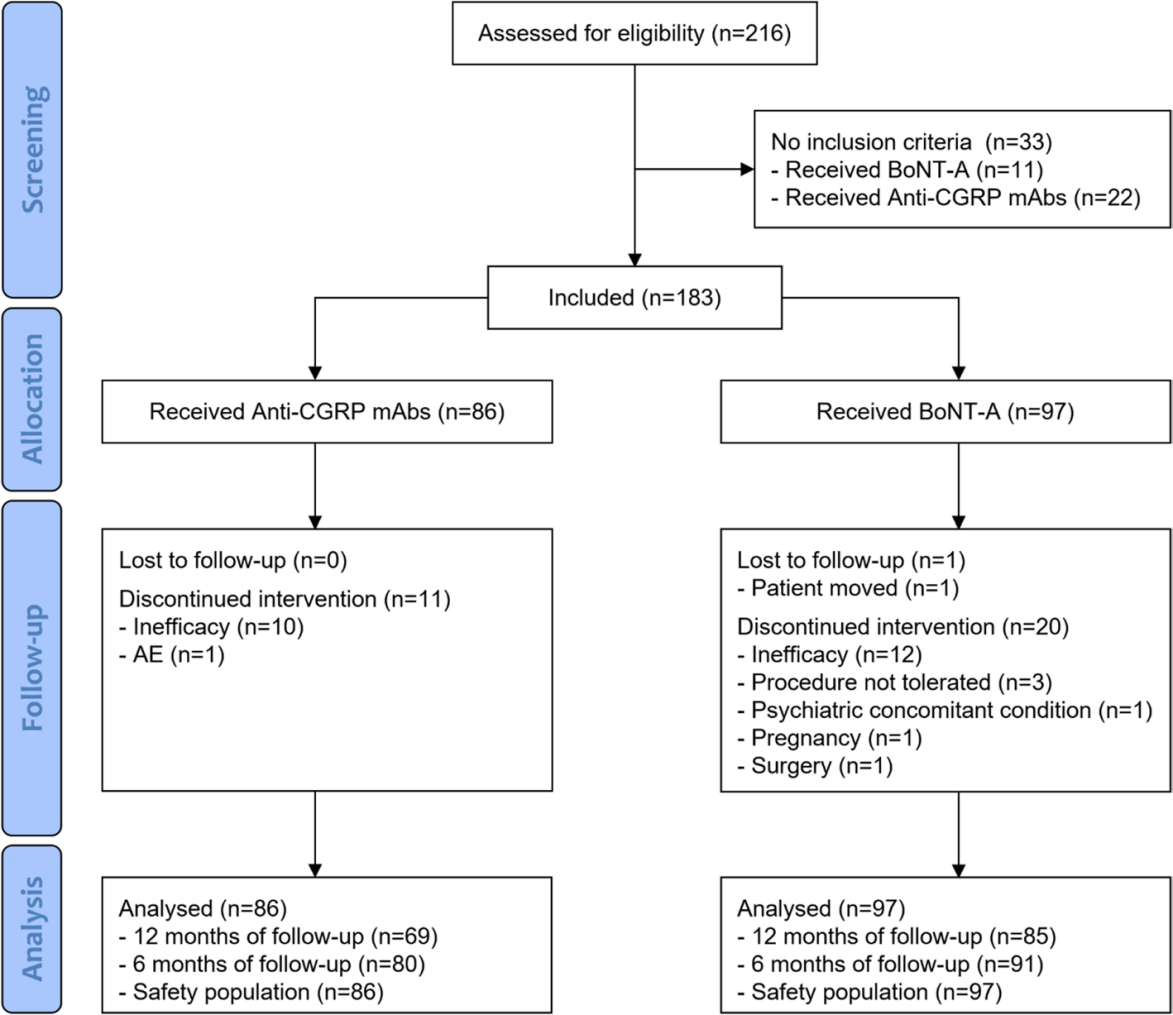
Study population selection and analysis

Up to 1 October 2023, a total of 216 potentially eligible patients had available data in internal databases of participating centers. After screening, 183 patients, 86 on Anti-CGRP mAbs and 97 on BoNT-A, met inclusion criteria to be included in the RAMO study. Of them, 69 patients receiving Anti-CGRP mAbs and 85 receiving BoNT-A had a follow-up time up to 12 months with available data on MHD and were included in the primary analysis (Fig. 1). Patients included in the BoNT-A arm started the medication between 2015 and 2019, while patients included in the Anti-CGRP mAbs received their first dose between 2019 and 2023. Among patients included in the Anti-CGRP mAbs group, 49 (57.0%) received galcanezumab, 22 (25.6%) erenumab, and 15 (17.4%) fremanezumab. The total population had a mean age of 46.1 years and a migraine history duration of 28.4 years, with non-significant differences between groups (Table 1). Data from seven patients (5 Anti-CGRP mAbs; 3 BoNT-A) with age at baseline more than 65 years were inadvertently collected; however, we considered these as minor protocol deviations and included patients in analyses. In the total population, females were more frequently represented (84.7%) with a significant difference between Anti-CGRP mAbs and BoNT-A patients (*p* = 0.023), being more represented in the latter group. MOH was reported in 79.8% of patients included in the overall populations, without significant differences between the study groups. Differently, TLS was present in 29.0% of included patients and more frequently reported in BoNT-A patients (*p* = 0.005). BoNT-A patients also presented significantly more comorbidities (in particular anxiety was the only subcategory significantly different; *p* < 0.001) and migraine concomitant medications (in particular tricyclic antidepressants was the only subcategory significantly different; *p* = 0.001) at baseline. Mean MHD at baseline was 20.3 days in the total population; however, BoNT-A patients had a significantly superior MHD mean at baseline compared to Anti-CGRP mAbs (21.7 vs. 18.7, respectively; *p* = 0.0029). Mean baseline MIDAS and MAM were 88.1 points and 20.9 single pill administrations, respectively, with non-significant between-group differences (*p* = 0.7089 and *p* = 0.1384, respectively). Complete baseline data are reported in Table 1.

**Table 1:** Population baseline characteristics

	Total (*n* = 183)	Anti-CGRP mAbs (*n *= 86)	BoNT-A (*n* = 97)	*p*-value
Age, years, mean (SD)	46.1 (11.3)	45.9 (12.7)	46.3 (10.0)	0.8612
Sex, female, n (%)	155 (84.7)	67 (77.9)	88 (90.7)	**0.023**
Center, n (%)				**< 0.001**
Milan	91 (49.7)	24 (27.9)	67 (69.1)	
Rome	92 (50.3)	62 (72.1)	30 (30.9)	
Migraine duration, years, mean (SD)	28.4 (11.3)	27.6 (12.6)	29.1 (10.0)	0.3379
MOH, n (%)	146 (79.8)	66 (76.4)	80 (82.5)	0.361
TLS, n (%)	53 (29.0)	16 (18.6)	37 (38.1)	**0.005**
Comorbidities, n (%)	102 (55.7)	38 (44.2)	64 (66.0)	**0.004**
Hypertension, n (%)	28 (15.3)	13 (15.1)	15 (15.5)	1.000
Depression, n (%)	27 (14.8)	10 (11.6)	17 (17.5)	0.301
Anxiety, n (%)	43 (23.5)	8 (9.3)	35 (36.1)	**< 0.001**
Epilepsy, n (%)	1 (0.6)	1 (1.2)	0 (0.0)	0.470
Cardiovascular, n (%)	7 (3.8)	2 (2.3)	5 (5.2)	0.450
Gastroenteric, n (%)	10 (5.5)	6 (7.0)	4 (4.1)	0.519
Chronic pain conditions, n (%)	4 (2.2)	2 (2.3)	2 (2.1)	1.000
Cancer, n (%)	3 (1.64)	1 (1.2)	2 (2.1)	1.000
Endocrine and metabolic, n (%)	11 (6.0)	7 (8.1)	4 (4.1)	0.353
Other, n (%)	24 (13.1)	11 (12.8)	13 (13.4)	1.000
Concomitant migraine medications, n (%)	89 (48.9)	25 (29.1)	64 (66.7)	**< 0.001**
Beta-blockers, n (%)	24 (13.1)	8 (9.3)	16 (16.5)	0.190
TCA, n (%)	22 (12.0)	3 (3.5)	19 (19.6)	**0.001**
Anti-Convulsant, n (%)	26 (14.2)	10 (11.6)	16 (16.5)	0.400
ARBs, n (%)	16 (8.7)	4 (4.7)	12 (12.4)	0.072
SSRI-SNRI, n (%)	31 (16.9)	11 (12.8)	20 (20.6)	0.173
Pizotifen, n (%)	2 (1.09)	0 (0.0)	2 (2.06)	0.499
SARI, n (%)	2 (1.09)	0 (0.0)	2 (2.06)	0.499
Flunarizine, n (%)	0 (0.0)	0 (0.0)	0 (0.0)	-
MHD, days, mean (SD)	20.3 (6.0)	18.7 (4.3)	21.7 (6.5)	**0.0029**
MIDAS, points, mean (SD)	88.1 (56.6)	84.6 (43.3)	91.3 (66.3)	0.7089
MAM, administrations, mean (SD)	20.9 (10.5)	18.6 (5.9)	22.9 (13.0)	0.1384

Among comorbidities, Other include allergic asthma, amenorrhea, atopic dermatitis, autoimmune hepatitis, autoimmune thyroid disease, chronic vein insufficiency, connectivitis, essential tremor, Helicobacter pylori, hepatitis B infection, hepatitis C infection, hip dysplasia, insomnia, osteoarthritis, pituitary adenoma, polycystic ovary, psoriasis, reduction surgery of the jaw, restless leg syndrome, scleroderma, tuberculosis test positivity, upper airways resistance syndrome, and urticaria

*Abbreviations: ARBs* Angiotensin receptor blockers, *BoNT-A *Onabotulinumtoxin-A, *CGRP *Calcitonin gene related peptide, *mAbs *Monoclonal antibodies, *MAM *Migraine acute medications, *MHD *Monthly headache days, *MIDAS *Migraine disability assessment test, *MOH *Medication overuse headache, *SARI *Serotonin antagonist and reuptake inhibitors, *SD *Standard deviation, *SNRI *Serotonin norepinephrine reuptake inhibitors, *SSRI *Selective serotonin reuptake inhibitors, *TCA *Tricyclic antidepressants, *TLS *Tension-like symptoms

### Efficacy outcomes

For the primary outcome of the study (i.e., MHD change from baseline at 12 months) patients receiving Anti-CGRP mAbs showed a mean reduction of 11.9 (SD 6.1) days compared to BoNT-A patients who had a 7.6 (SD 8.5) days reduction. The unadjusted mean difference was -4.4 days with a 95%CI ranging from -6.8 to -2.0 (*p* = 0.0002). After adjusting for potential baseline confounders, marginal mean difference resulted -6.2 days with a 95%CI from -9.2 to -4.7 (*p* < 0.001). Similar results were obtained in the propensity score matched model (mean difference -7.6 days; 95%CI from -10.9 to -4.3; *p* < 0.001) (Table 2).

**Fig. 2:**
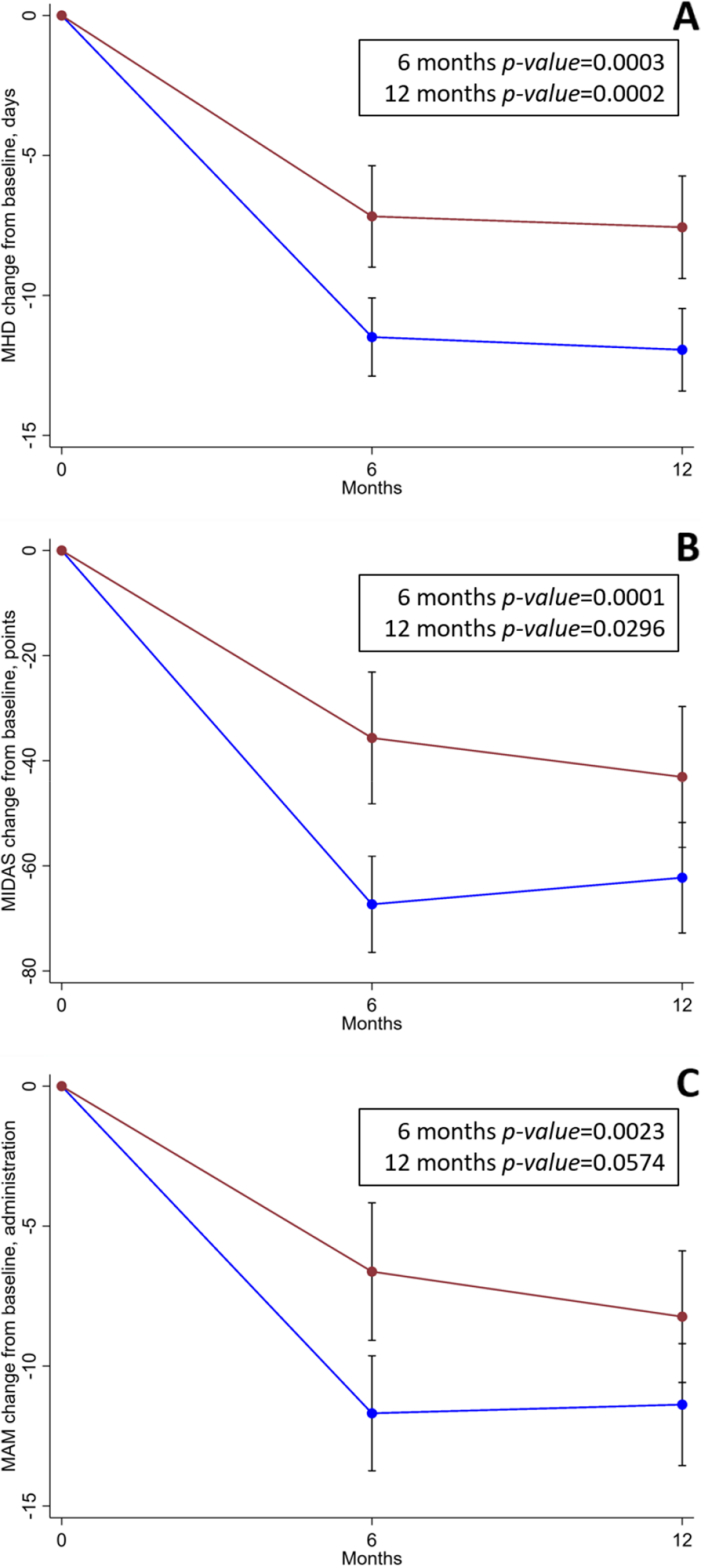
Efficacy outcomes. Mean changes form baseline for **A** MHD, **B** MIDAS and **C** MAM at 6 and 12 months of follow-up. Blue lines indicate Anti-CGRP mAbs; red lines indicate BoNT-A; error bars represent 95%CI. Reported *p*-values are those from the unadjusted analysis. Abbreviations: MAM = migraine acute medications; MHD = monthly headache days; MIDAS = migraine disability assessment test

Similar results were obtained with the MHD change from baseline at 6 months (*p* = 0.0002) (Table 2). Anti-CGRP mAbs showed a significantly different MIDAS reduction compared to BoNT-A at 6 (unadjusted mean difference -31.7 points; 95%CI from -47.4 to -15.9) and 12 (unadjusted mean difference -19.2 points; 95%CI from -36.4 to -1.9; *p* = 0.0296) months (Table 2). Similarly, Anti-CGRP mAbs significantly reduced MAM more than BoNT-A at 6 months (unadjusted mean difference -5.1 administrations; 95%CI from -8.3 to -1.8) and at limits of statistical non-significance at 12 months (unadjusted mean difference -3.1 administrations; 95%CI from -6.4 to 0.1). These results were substantially confirmed after adjusting for potential baseline confounders and in the propensity score matched model apart from MIDAS reduction at 12 months, which resulted non-significantly different (Table 2). The changes from baseline for MHD, MIDAS, and MAM are described in Fig. 2. Response rate was significantly superior in the Anti-CGRP mAbs group compared to BoNT-A at 50% and 75% levels of response both at 6 and 12 months, while non-significant differences were observed at 100% level of response at both time points. The adjusted logistic regression model confirmed the results from the unadjusted analysis. Complete results on response rate are provided in Table 3.

Erenumab, fremanezumab, and galcanezumab showed substantial comparability in their effectiveness without significant differences in-between different Anti-CGRP mAbs (Supplementary material S2).

**Table 2:** Efficacy outcomes

	Anti-CGRP mAbs	BoNT-A	Unadjusted Mean difference (95%CI)	*p*-value	Adjusted Mean difference (95%CI)	*p*-value	Propensity score matched Mean difference (95%CI)	*p*-value
**Primary outcome**								
MHD 12 months change from baseline, mean (SD)	-11.9 (6.1)	-7.6 (8.5)	-4.4 (-6.8 to -2.0)	**0.0002**	-6.2 (-9.2 to -4.7)	**< 0.001**	-7.6 (-10.9 to -4.3)	**< 0.001**
	*n* = 69	*n* = 85						
**Secondary outcomes**								
MHD 6 months change from baseline, mean (SD)	-11.5 (6.3)	-7.2 (8.7)	-4.3 (-6.6 to -2.0)	**0.0003**	-7.1 (-9.5 to -4.7)	**< 0.001**	-6.7 (-11.2 to -2.2)	**0.004**
	*n* = 80	*n* = 91						
MIDAS 12 months change from baseline, mean (SD)	-62.3 (43.8)	-43.1 (59.8)	-19.2 (-36.4 to -1.9)	**0.0296**	-18.5 (-37.7 to 0.7)	0.059	-7.3 (-41.6 to 27.1)	0.678
	*n* = 69	*n* = 79						
MIDAS 6 months change from baseline, mean (SD)	-67.3 (41.1)	-35.7 (60.2)	-31.7 (-47.4 to -15.9)	**0.0001**	-34.3 (-52.7 to -16.0)	**< 0.001**	-30.0 (-51.5 to -8.4)	**0.006**
	*n* = 80	*n* = 91						
MAM 12 months change from baseline, mean (SD)	-11.4 (9.1)	-8.2 (10.8)	-3.1 (-6.4 to 0.1)	0.0574	-5.6 (-9.3 to -2.0)	**0.003**	-8.1 (-11.8 to -4.3)	**< 0.001**
	*n* = 69	*n* = 85						
MAM 6 months change from baseline, mean (SD)	-11.7 (9.2)	-6.6 (11.8)	-5.1 (-8.3 to -1.8)	**0.0023**	-7.6 (-11.4 to -3.9)	**< 0.001**	**-**7.4 (-10.5 to -4.5)	**< 0.001**
	*n* = 80	*n* = 91						

The number of patients included in each analysis is reported under the corresponding results

* Abbreviations: 95%CI* 95% confidence interval, *BoNT-A* Onabotulinumtoxin-A, *CGRP* Calcitonin gene related peptide, *mAbs* Monoclonal antibodies, *MAM* Migraine acute medications, *MHD* Monthly headache days, *MIDAS* Migraine disability assessment test, *SD* Standard deviation

**Table 3:** Responder analysis

	Anti-CGRP mAbs (*n* = 69)	BoNT-A (*N *= 85)	*p*-value	Adjusted OR (95%CI)	*p*-value
Responders at 12 months					
50%, *n* (%)	49 (71.0)	33 (38.8)	**< 0.001**	8.6 (3.4 to 22.1)	**< 0.001**
75%, *n* (%)	24 (34.8)	9 (10.6)	**< 0.001**	8.3 (2.6 to 27.0)	**< 0.001**
100%, *n* (%)	2 (2.9)	0 (0.0)	0.199	NA	NA
Responders at 6 months					
50%, *n* (%)	53 (76.8)	27 (31.8)	**< 0.001**	18.2 (6.4 to 52.1)	**< 0.001**
75%, *n* (%)	30 (43.5)	11 (12.9)	**< 0.001**	12.5 (3.8 to 41.1)	**< 0.001**
100%, *n* (%)	3 (4.4)	0 (0.0)	0.088	NA	NA

Responders were defined as patients achieving a reduction in MHD of 50%, 75%, and 100% at 6 and 12 months of follow-up compared to baseline

Responder analysis was performed on the population included in the primary outcome analysis

*Abbreviations: 95%CI *95% confidence interval, *BoNT-A *Onabotulinumtoxin-A, *CGRP *Calcitonin gene related peptide, *mAbs *Monoclonal antibodies, *MHD *Monthly headache days, *NA *Not applicable, *OR *Odds ratio

### Safety outcomes

Safety was investigated in the total population. Both investigated treatments showed a comparable safety profile and no patient reported any SAE. Only 1 patient receiving fremanezumab discontinued the treatment due to grade 2 arthralgia, for which he started to use NSAIDS. He had pre-existing hypertension, hypothyroidism, and osteoarthritis as concomitant conditions. After treatment discontinuation arthralgia resolved without needing medications in few weeks. Treatment discontinuations due to other reasons were similar between Anti-CGRP mAbs and BoNT-A patients (*p* = 0.113), with discontinuations occurring mainly for inefficacy in 11.6% and 12.4% of patients, respectively. Safety outcomes are reported in Table 4.

**Table 4:** Safety outcomes

	Anti-CGRP mAbs (*n* = 86)	BoNT-A (*n* = 97)	*p*-value
SAE, *n* (%)	0 (0.0)	0 (0.0)	-
Discontinuation due to AE, *n* (%)	1 (1.2)	0 (0.0)	0.470
Arthralgia	1 (1.2)	0 (0.0)	-
Discontinuation due to other reasons, *n* (%)	10 (11.6)	20 (20.6)	0.113
Inefficacy	10 (11.6)	12 (12.4)	-
Injection procedure not tolerated	0 (0.0)	3 (3.1)	-
Psychiatric concomitant conditions	0 (0.0)	1 (1.0)	-
Pregnancy	0 (0.0)	1 (1.0)	-
Surgery	0 (0.0)	1 (1.0)	-
Patient moved	0 (0.0)	1 (1.0)	

*Abbreviations: AE *Adverse events, *BoNT-A *Onabotulinumtoxin-A, *CGRP *Calcitonin gene related peptide, *mAbs *Monoclonal antibodies, *SAE *Serious adverse events

## Discussion

In the RAMO study, we observed a significantly higher magnitude of effectiveness in all investigated outcomes for Anti-CGRP mAbs compared to BoNT-A. In particular, the difference was significant both at 6 and at 12 months of follow-up in the unadjusted analysis. In our cohort, BoNT-A patients showed higher MHDs at baseline, as well as reporting more frequently TLS, anxiety, and concomitant preventive medication. In other words, they showed some characteristics of a more severe migraine condition compared to Anti-CGRP mAbs patients. To reduce these potentially relevant baseline confounding factors we produced an adjusted analysis, which substantially confirmed the unadjusted results. These findings paralleled what we observed for MIDAS and MAM, further confirming the results on MHD, while both treatments showed a good and generally comparable safety profile. However, the mean difference for MIDAS at 12 months resulted non-significantly different in both the adjusted model and in the propensity score matched model, possibly indicating that, even with a superior reduction in MHD with Anti-CGRP mAbs, the reduction in disability might be comparable in-between treatments at 12 months of follow-up.

In our cohort, the observed effectiveness of BoNT-A was similar to that showed by the pivotal randomized clinical trials (RCT) investigating BoNT-A against placebo, PREEMPT 1 and PREEMPT 2, which reported a MHD reduction of -7.8 and -9.0 days in the BoNT-A arm, respectively, at 6 months of follow-up [8, 39]. A Cochrane meta-analysis comparing BoNT-A to placebo showed results consistent to other meta-analyses and included trials with absolute reductions in MHD similar to ours in the BoNT-A arm, although the quality of the evidence was in general low [6]. Conversely, when comparing absolute MHD reductions in Anti-CGRP mAbs arms of RCTs including CM patients to our results, we generally observed larger improvements. Indeed, RCTs showed absolute mean MHD reductions of -4.3 and -4.1 days for fremanezumab and -4.8 and -4.6 for galcanezumab, while for erenumab a reduction in monthly migraine days of -5.1 was observed [18, 20, 40, 41].

A meta-analysis aimed to perform and indirect comparison of Anti-CGRP mAbs and BoNT-A efficacy in RCTs showed comparable efficacy of the two treatments, with a slightly better efficacy and safety for Anti-CGRP mAbs [37]. Another meta-analysis showed absolute MHD reductions with BoNT-A treatment comparable to our findings in patients with CM and depression; the absolute MIDAS reduction was also comparable [42].

However, the majority of these trials assessed efficacy outcomes after 3 months of treatment, and only two studies on BoNT-A had a follow-up time of 4 and 6 months [9, 42–44], respectively, consistently limiting the comparability of those result to ours. Studies comparing different treatments, in particular in meta-analyses, present some limitations, such as different study duration, definitions of AE, and use of different outcome measures (e.g., migraine attacks, migraine days, headache days, etc.) [44]. The present study is in line with previous real-world studies on Anti-CGRP mAbs showing a more pronounced benefit compared to RCTs. In CM patients, they showed a MHD reduction at 12 months of -15 days for fremanezumab, -11.9 days for galcanezumab, and -12.8 days for erenumab. Our response rate was also generally comparable to what observed in other studies [45–49].

The mechanisms of action of the two therapeutic approaches might explain the different outcomes. Fremanezumab directly block the CGRP pathway and is believed to act on Aδ fibers and given the common pharmacodynamical target, this action could be a class effect shared also with erenumab, galcanezumab, and eptinezumab [50]. Conversely, BoNT-A indirectly inhibits the release of neurotransmitters by C fibers by cleaving the SNAP-25 protein, which allows anchorage of vesicles in the presynaptic cell membrane [51, 52]. Both treatments probably exert a primary peripheral action on trigeminal targets associated with a secondary modulation of central sensitization. It is possible that the direct inhibition of CGRP has a more powerful efficacy in migraine prevention as it also influences the course of acute attacks, as demonstrated by the acute action of CGRP-receptor antagonist (i.e., gepants) [53]. Nevertheless, since the two treatments have different pharmacological targets, combinatorial therapy might be considered to achieve better CM control as also proposed by other authors [52, 54, 55].

 Regardless, our results are still confirmatory of the real-world effectiveness of both Anti-CGRP mAbs and BoNT-A and showed a sustained response up to 12 months of the initial 6-months response, while our observed superiority of Anti-CGRP mAbs should be confirmed in future comparative studies. No direct comparisons between Anti-CGRP mAbs and BoNT-A for migraine are available. This is partly due to the difficulty of conducting double-blinded studies in this context due to the two different ways of administration. For this reason, future randomized trials comparing these two medications should implement accurate strategies for double-dummies and masking in order to assure blinding. The only direct comparison evaluating an Anti-CGRP mAb to another prophylactic treatment is the Head-to-head study of erenumab against topiramate - Migraine study to assess tolerability and efficacy in a patient-centred setting (HER-MES). The HER-MES randomized controlled trial was a double-blinded double-dummy study conducted in Germany, enrolling both episodic and CM patients, whose results showed a superior efficacy and tolerability for erenumab compared to topiramate [56].

Besides RCTs, observational real-world studies should be fostered since the approach to the patient might be different in terms of attention and clinical setting between randomized trials and the usual practice, contributing to the contextual effect, which could be considerably relevant as previously observed in a meta-analysis on randomized trials on Anti-CGRP mAbs [57]. Thus, the possible contextual effect in randomized trials included in studies indirectly comparing Anti-CGRP mAbs to BoNT-A as well as the expectations of patients receiving Anti-CGRP mAbs together with the different periods in which patients received BoNT-A in our cohort (i.e., before Anti-CGRP mAbs were introduced in the market) might explain the differences between our results and those of previous meta-analyses, indicating the need for further real-world evidence in this setting [37, 57].

In our study, both treatments showed a favorable and generally comparable safety profile. The discontinuation rate due to inefficacy was also comparable. One patient receiving fremanezumab discontinued the treatment for arthralgia and three patients receiving BoNT-A interrupted the treatment due to intolerance to injection procedure. The latter event is not unexpected since BoNT-A administration requests several injections and might be associated with local and transitory effects (e.g., muscular weakness, pain, inflammation) [58]. However, albeit patient injection-related discomfort might be an additional barrier to BoNT-A use, the quarterly visits to the health professional without the need to take daily medication may enhance compliance with treatment, especially in patients who tolerate the procedure [5].

Still, BoNT-A clinical relevance remains indisputable and its cost-effectiveness profile resulted superior to that of other oral prophylactic medications in terms of reduced emergency department admissions, urgent care visits, and headache-related hospitalizations [58–60]. Anti-CGRP mAbs showed considerable health economic savings and socioeconomic gains but also present elevated costs, while other authors suggested that if BoNT-A costs were lower it would likely be recommended as an early preventive treatment [6, 61]. Since treatment access and cost-effectiveness evaluations may differ greatly between different nations, direct comparisons of the cost-effectiveness of BoNT-A and Anti-CGRP mAbs are needed in different socio-economic settings. BoNT-A remains a treatment of choice in the CM population with contraindications to mAbs treatment, such as uncontrolled hypertension, previous heart attack, or cerebral and cardiovascular diseases. Moreover, the experience with BoNT-A in the elderly confirms the efficacy and tolerability in this population [62]. A recent cohort study in UK also showed the safety of BoNT-A during pregnancy in women with CM [63]. These findings encourage the use of BoNT-A in frail categories and patients with unmet needs. Finally, but not less important, as the range of therapeutic possibilities in migraine is expanding, the patients are invited to express their preferences. The process on treatment selection must include common decision-making. In this scenario, we shall not neglect the preference some patients can express for a hospital based, regularly scheduled, and local treatment that has no pharmaceutical interaction or systemic side effects [64, 65].

### Limitations

Our study has limitations. The retrospective design with data from two different time periods coming from databases not initially designed for this specific study may be a source of bias. However, we included stringent inclusion criteria, excluding patients who received both investigated treatments, thus limiting the potential bias on the effectiveness estimates of BoNT-A non-responders who then switched to Anti-CGRP mAbs. Also, we could not differentiate monthly migraine days from MHD and the different MAM (e.g., triptans, NSAIDS), since in patient-self reported diaries in use in our clinical practice this detail of information is not collected. Future prospective studies should implement such different outcomes, which could be relevant in the context of CM treatment. Differences between groups at baseline (especially MHD, anxiety, and TLS) could indicate that patients included in the BoNT-A group might a have a more severe migraine condition. Even though we confirmed our unadjusted results after correcting for baseline differences, residual bias could not be excluded. The Anti-CGP mAbs arm does not include eptinezumab since at the time of our data collection this dug was not available in the Italian market. Eventually, the role of contextual effect and major expectations from Anti-CGRP mAbs might have contributed to their superior effectiveness, although the long-term follow-up might have limited the associated bias.

## Conclusion

In our sample of patients with CM Anti-CGRP mAbs were superior to BoNT-A in reducing MHD, MIDAS, and MAM at 6 and 12 months, with a comparable safety profile. However, BoNT-A remains a valuable and suitable option for patients with CM, in particular for those who have comorbidities contraindicating the use of Anti-CGRP mAbs and for frail or particular categories of patients who are candidates to local therapy with limited risk of systemic administration. Moreover, according to their different mechanisms of action, the possible combination of Anti-CGRP mAbs to BoNT-A could be considered one more option for resistant/refractory patients so that to increase their therapeutic potential. The results of this study could provide new insights for treatment choice in clinical practice, according to effectiveness, safety, and cost-benefit of these drugs. Further studies are needed to confirm our findings.

### Supplementary Information

**Additional file 1:** **Supplementary material S1. **STROBE Statement Checklist.** Supplementary material S2. **Anti-CGRP mAbs subgroup analysis. **Table S1. **Population baseline characteristics by different anti-CGRP mAb. **Table S2. **Efficacy outcomes by different anti-CGRP mAb.

## Data Availability

The datasets used and/or analysed during the current study are available from the corresponding author on reasonable request.

## Abbreviations

95%CI: 95% confidence interval
AE: Adverse events
ANOVA: Analysis of variance
ARBs: Angiotensin receptor blockers
BoNT-A: OnabotulinumtoxinA
CGRP: Calcitonin gene-related peptide
CM: Chronic migraine
CTCAE: Common Terminology Criteria for Adverse Events
HER-MES: Head-to-head study of erenumab against topiramate - Migraine study to assess tolerability and efficacy in a patient-centred setting
mAbs: Monoclonal antibodies
MAM: Monthly migraine acute medications
MedDRA: Medical Dictionary for Regulatory Activities Terminology
MHD: Monthly headache days
MIDAS: Migraine disability assessment test
MOH: Medication overuse headache
OR: Odds ratio
PREEMPT: Phase III Research Evaluating Migraine Prophylaxis Therapy
RAMO: Real-world effectiveness of Anti-CGRP Monoclonal antibodies compared to OnabotulinumtoxinA
RCT: Randomized controlled trials
REDCap: Research Electronic Data Capture
SAE: Serious adverse events
SARI: Serotonin antagonist and reuptake inhibitors
SNRI: Serotonin norepinephrine reuptake inhibitors
SSRI: Selective serotonin reuptake inhibitors
SD: Standard deviation
STROBE: STrengthening the Reporting of OBservational studies in Epidemiology
TCA: Tricyclic antidepressants
TLS: Tension-like symptoms
