# Supplementary material for: Real-world effectiveness of Anti-CGRP monoclonal antibodies compared to OnabotulinumtoxinA (RAMO) in chronic migraine: a retrospective, observational, multicenter, cohort study
Source: J Headache Pain. 2024 Feb 2;25(1):14. doi: 10.1186/s10194-024-01721-6 (PMC10836018; doi:10.1186/s10194-024-01721-6)
Supplement: Supplementary file 1 — Additional file 1: Supplementary material S1. STROBE Statement Checklist. Supplementary material S2. Anti-CGRP mAbs subgroup analysis. Table S1. Population baseline characteristics by different anti-CGRP mAb. Table S2. Efficacy outcomes by different anti-CGRP mAb. [file 10194_2024_1721_MOESM1_ESM.docx]

**Real-world effectiveness of Anti-CGRP Monoclonal antibodies compared to OnabotulinumtoxinA (RAMO) in chronic migraine: a retrospective, observational, multicenter, cohort study**

Licia Grazzi*; Riccardo Giossi*; Danilo Antonio Montisano; Mattia Canella; Marilena Marcosano; Claudia Altamura; Fabrizio Vernieri

* Licia Grazzi and Riccardo Giossi equally contributed to this work and shared first co-authorship.

**SUPPLEMENTARY MATERIAL**

**Page 2 Supplementary material S1. STROBE Statement Checklist**

**Page 4 Supplementary material S2. Anti-CGRP mAbs subgroup analysis**

**Supplementary material S1. STROBE Checklist**

Checklist of items that should be included in reports of ***cohort studies***

|  | Item No | Recommendation | Page |
| --- | --- | --- | --- |
| **Title and abstract** | 1 | (*a*) Indicate the study’s design with a commonly used term in the title or the abstract | Title Page |
|  |  | (*b*) Provide in the abstract an informative and balanced summary of what was done and what was found | 2 |
| Introduction | | |  |
| Background/rationale | 2 | Explain the scientific background and rationale for the investigation being reported | 4 |
| Objectives | 3 | State specific objectives, including any prespecified hypotheses | 5 |
| Methods | | |  |
| Study design | 4 | Present key elements of study design early in the paper | 5 |
| Setting | 5 | Describe the setting, locations, and relevant dates, including periods of recruitment, exposure, follow-up, and data collection | 5 |
| Participants | 6 | (*a*) Give the eligibility criteria, and the sources and methods of selection of participants. Describe methods of follow-up | 5 |
|  |  | (*b*) For matched studies, give matching criteria and number of exposed and unexposed | NA |
| Variables | 7 | Clearly define all outcomes, exposures, predictors, potential confounders, and effect modifiers. Give diagnostic criteria, if applicable | 5-6 |
| Data sources/ measurement | 8* | For each variable of interest, give sources of data and details of methods of assessment (measurement). Describe comparability of assessment methods if there is more than one group | 5-6 |
| Bias | 9 | Describe any efforts to address potential sources of bias | 5-7 |
| Study size | 10 | Explain how the study size was arrived at | 6-7 |
| Quantitative variables | 11 | Explain how quantitative variables were handled in the analyses. If applicable, describe which groupings were chosen and why | 6-7 |
| Statistical methods | 12 | (*a*) Describe all statistical methods, including those used to control for confounding | 6-7 |
|  |  | (*b*) Describe any methods used to examine subgroups and interactions | 6-7 |
|  |  | (*c*) Explain how missing data were addressed | 6-7 |
|  |  | (*d*) If applicable, explain how loss to follow-up was addressed | NA |
|  |  | (*e*) Describe any sensitivity analyses | 6-7 |
| Results | | |  |
| Participants | 13* | (a) Report numbers of individuals at each stage of study—eg numbers potentially eligible, examined for eligibility, confirmed eligible, included in the study, completing follow-up, and analysed | 7-8, Figure 1, Table 1, Table 2 |
|  |  | (b) Give reasons for non-participation at each stage | 7-8, Figure 1 |
|  |  | (c) Consider use of a flow diagram | Figure 1 |
| Descriptive data | 14* | (a) Give characteristics of study participants (eg demographic, clinical, social) and information on exposures and potential confounders | 7-8, Table 1 |
|  |  | (b) Indicate number of participants with missing data for each variable of interest | All Tables |
|  |  | (c) Summarise follow-up time (eg, average and total amount) | 7-8, Figure 1, Table 2 |
| Outcome data | 15* | Report numbers of outcome events or summary measures over time | 10-14, Table 2, Table 4 |
| Main results | 16 | (*a*) Give unadjusted estimates and, if applicable, confounder-adjusted estimates and their precision (eg, 95% confidence interval). Make clear which confounders were adjusted for and why they were included | 10-14, Table 2, Table 3 |
|  |  | (*b*) Report category boundaries when continuous variables were categorized | 10, Table 2 |
|  |  | (*c*) If relevant, consider translating estimates of relative risk into absolute risk for a meaningful time period | NA |
| Other analyses | 17 | Report other analyses done—eg analyses of subgroups and interactions, and sensitivity analyses | 10-11, Table 2, Table 3, Table 4, Table S2 |
| Discussion | | |  |
| Key results | 18 | Summarise key results with reference to study objectives | 14-17 |
| Limitations | 19 | Discuss limitations of the study, taking into account sources of potential bias or imprecision. Discuss both direction and magnitude of any potential bias | 17-18 |
| Interpretation | 20 | Give a cautious overall interpretation of results considering objectives, limitations, multiplicity of analyses, results from similar studies, and other relevant evidence | 14-18 |
| Generalisability | 21 | Discuss the generalisability (external validity) of the study results | 14-18 |
| Other information | | |  |
| Funding | 22 | Give the source of funding and the role of the funders for the present study and, if applicable, for the original study on which the present article is based | 21 |

**Supplementary material S2. Anti-CGRP mAbs subgroup analysis**

| **Table S1. Population baseline characteristics by different anti-CGRP mAb** | | | | |
| --- | --- | --- | --- | --- |
|  | **Galcanezumab**  **(n=49)** | **Erenumab (n=22)** | **Fremanezumab**  **(n=15)** | **p-value** |
| Age, years, mean (SD) | 43.0 (12.5) | 46.2 (11.2) | 54.8 (11.7) | **0.0170** |
| Sex, female, n (%) | 41 (83.7) | 14 (63.6) | 12 (80.0) | 0.166 |
| Center, n (%) |  |  |  | 0.422 |
| Milan | 11 (22.5) | 8 (36.4) | 5 (33.3) |  |
| Rome | 38 (77.6) | 14 (63.6) | 10 (66.7) |  |
| Migraine duration, years, mean (SD) | 25.6 (12.3) | 27.1 (1.5) | 34.9 (13.2) | 0.0828 |
| MOH, n (%) | 38 (77.6) | 16 (72.7) | 12 (80.0) | 0.858 |
| TLS, n (%) | 9 (18.4) | 5 (22.7) | 2 (13.3) | 0.770 |
| Comorbidities, n (%) | 20 (40.8) | 8 (36.4) | 10 (66.7) | 0.146 |
| Hypertension, n (%) | 6 (12.2) | 3 (13.6) | 4 (26.7) | 0.384 |
| Depression, n (%) | 6 (12.2) | 4 (18.2) | 0 (0.0) | 0.233 |
| Anxiety, n (%) | 7 (14.3) | 1 (4.6) | 0 (0.0) | 0.168 |
| Epilepsy, n (%) | 1 (2.0) | 0 (0.0) | 0 (0.0) | 0.682 |
| Cardiovascular, n (%) | 1 (2.0) | 0 (0.0) | 1 (6.7) | 0.409 |
| Gastroenteric, n (%) | 4 (8.2) | 0 (0.0) | 2 (13.3) | 0.261 |
| Chronic pain conditions, n (%) | 0 (0.0) | 1 (4.6) | 1 (6.7) | 0.236 |
| Cancer, n (%) | 0 (0.0) | 0 (0.0) | 1 (6.7) | 0.091 |
| Endocrine and metabolic, n (%) | 3 (6.1) | 1 (4.6) | 3 (20.0) | 0.177 |
| Other, n (%) | 5 (10.2) | 3 (13.6) | 3 (20.0) | 0.604 |
| Concomitant migraine medications, n (%) | 15 (30.6) | 5 (22.7) | 5 (33.3) | 0.734 |
| Beta-blockers, n (%) | 7 (14.3) | 1 (4.6) | 0 (0.0) | 0.168 |
| TCA, n (%) | 0 (0.0) | 2 (9.1) | 1 (6.7) | 0.118 |
| Anti-Convulsant, n (%) | 7 (14.3) | 1 (4.6) | 2 (13.3) | 0.484 |
| ARBs, n (%) | 2 (4.1) | 0 (0.0) | 2 (13.3) | 0.161 |
| SSRI-SNRI, n (%) | 6 (12.2) | 2 9.1) | 3 (20.0) | 0.612 |
| Pizotifen, n (%) | 0 (0.0) | 0 (0.0) | 0 (0.0) | NA |
| SARI, n (%) | 0 (0.0) | 0 (0.0) | 0 (0.0) | NA |
| Flunarizine, n (%) | 0 (0.0) | 0 (0.0) | 0 (0.0) | NA |
| MHD, days, mean (SD) | 19.6 (4.9) | 17.3 (4.9) | 18.0 (4.0) | 0.0818 |
| MIDAS, points, mean (SD) | 88.7 (43.3) | 75.3 (48.3) | 84.8 (43.6) | 0.2301 |
| MAM, administrations, mean (SD) | 19.2 (6.1) | 17.4 (6.3) | 18.2 (4.5) | 0.2298 |
| Abbreviations: ARBs=angiotensin receptor blockers; BoNT-A=Onabotulinumtoxin-A; CGRP=calcitonin gene related peptide; mAbs=monoclonal antibodies; MAM=migraine acute medications; MHD=monthly migraine headache days; MIDAS=migraine disability assessment test; MOH=medication overuse headache; NA=not applicable; SARI=serotonin antagonist and reuptake inhibitors; SD=standard deviation, SNRI=serotonin norepinephrine reuptake inhibitors; SSRI=selective serotonin reuptake inhibitors; TCA=tricyclic antidepressants; TLS=tension-like symptoms.  Among comorbidities, Other include allergic asthma, amenorrhea, atopic dermatitis, autoimmune hepatitis, autoimmune thyreopathy, chronic vein insufficiency, connectivitis, essential tremor, Helicobacter pylori, hepatitis B infection, hepatitis C infection, hip dysplasia, insomnia, osteoarthritis, pituitary adenoma, polycystic ovary, psoriasis, reduction surgery of the jaw, restless leg syndrome, scleroderma, tuberculosis test positivity, upper airways resistance syndrome, and urticaria. | | | | |

| **Table S2. Efficacy outcomes by different anti-CGRP mAb** | | | | |
| --- | --- | --- | --- | --- |
|  | **Galcanezumab**  **(n=49)** | **Erenumab (n=22)** | **Fremanezumab**  **(n=15)** | **p-value** |
| **Primary outcome** |  |  |  |  |
| MHD 12 months change from baseline, mean (SD) | -13.0 (6.1) | -11.3 (6.1) | -9.3 (5.7) | 0.2333 |
|  | n=39 | n=19 | n=11 |  |
| **Secondary outcomes** |  |  |  |  |
| MHD 6 months change from baseline, mean (SD) | -12.2 (6.2) | -10.1 (7.2) | -11.4 (5.0) | 0.2754 |
|  | n=45 | n=21 | n=14 |  |
| MIDAS 12 months change from baseline, mean (SD) | -64.4 (44.4) | -57.4 (40.6) | -63.0 (50.1) | 0.8893 |
|  | n=39 | n=19 | n=11 |  |
| MIDAS 6 months change from baseline, mean (SD) | -71.6 (41.7) | -53.9 (39.8) | -73.8 (39.8) | 0.1657 |
|  | n=45 | n=21 | n=14 |  |
| MAM 12 months change from baseline, mean (SD) | -11.9 (9.7) | -11.0 (8.1) | -10.1 (8.8) | 0.9108 |
|  | n=39 | n=19 | n=11 |  |
| MAM 6 months change from baseline, mean (SD) | -12.5 (7.0) | -8.3 (14.0) | -14.2 (4.7) | 0.2076 |
|  | n=45 | n=21 | n=14 |  |
| Responders at 12 months ^a^ |  |  |  |  |
| 50%, n (%) | 29 (74.4) | 13 (68.4) | 7 (63.6) | 0.754 |
| 75%, n (%) | 16 (41.0) | 5 (26.3) | 3 (27.3) | 0.462 |
| 100%, n (%) | 1 (2.6) | 1 (5.3) | 0 (0.0) | 0.697 |
| Responders at 6 months ^a^ |  |  |  |  |
| 50%, n (%) | 30 (76.9) | 14 (73.7) | 9 (81.8) | 0.878 |
| 75%, n (%) | 15 (38.5) | 7 (36.8) | 8 (72.7) | 0.102 |
| 100%, n (%) | 1 (2.6) | 1 (5.3) | 1 (9.1) | 0.628 |
| Abbreviations: 95%CI=95% confidence interval; BoNT-A=Onabotulinumtoxin-A; CGRP=calcitonin gene related peptide; mAbs=monoclonal antibodies; MAM=migraine acute medications; MHD=monthly migraine headache days; MIDAS=migraine disability assessment test; NA=not applicable; OR=odds ratio; SD=standard deviation.  The number of patients included in each analysis is reported under the corresponding results. Responders were defined as patients achieving a reduction in MHD of 50%, 75%, and 100% at 6 and 12 months of follow up compared to baseline.  ^a^ Responder analysis was performed on the population included in the primary outcome analysis. | | | | |
